# Use of Post-transplant Cyclophosphamide Treatment to Build a Tolerance Platform to Prevent Liquid and Solid Organ Allograft Rejection

**DOI:** 10.3389/fimmu.2021.636789

**Published:** 2021-03-02

**Authors:** Casey O. Lightbourn, Dietlinde Wolf, Sabrina N. Copsel, Ying Wang, Brent J. Pfeiffer, Henry Barreras, Cameron S. Bader, Krishna V. Komanduri, Victor L. Perez, Robert B. Levy

**Affiliations:** ^1^Department of Microbiology and Immunology, Miller School of Medicine, University of Miami, Miami, FL, United States; ^2^Sylvester Comprehensive Cancer Center, Miller School of Medicine, University of Miami, Miami, FL, United States; ^3^Department of Ophthalmology, Miller School of Medicine, University of Miami, Miami, FL, United States; ^4^Department of Pediatrics, Miller School of Medicine, University of Miami, Miami, FL, United States; ^5^Department of Medicine, Miller School of Medicine, University of Miami, Miami, FL, United States; ^6^Foster Center for Ocular Immunology at Duke Eye Center, Duke University, Durham, NC, United States

**Keywords:** cyclophosphamide, Treg, corneal transplantation, hematopoietic stem cell transplantation, tolerance

## Abstract

Corneal transplantation (CT) is the most frequent type of solid organ transplant (SOT) performed worldwide. Unfortunately, immunological rejection is the primary cause of graft failure for CT and therefore advances in immune regulation to induce tolerance remains an unmet medical need. Recently, our work and others in pre-clinical studies found that cyclophosphamide (Cy) administered after (“post-transplant,” PTCy) hematopoietic stem cell transplantation (HSCT), i.e., liquid transplants is effective for graft vs. host disease prophylaxis and enhances overall survival. Importantly, within the past 10 years, PTCy has been widely adopted for clinical HSCT and the results at many centers have been extremely encouraging. The present studies found that Cy can be effectively employed to prolong the survival of SOT, specifically mouse corneal allografts. The results demonstrated that the timing of PTCy administration is critical for these CT and distinct from the kinetics employed following allogeneic HSCT. PTCy was observed to interfere with neovascularization, a process critically associated with immune rejection of corneal tissue that ensues following the loss of ocular “immune privilege.” PTCy has the potential to delete or directly suppress allo-reactive T cells and treatment here was shown to diminish T cell rejection responses. These PTCy doses were observed to spare significant levels of CD4^+^ FoxP3^+^ (Tregs) which were found to be functional and could readily receive stimulating signals leading to their *in vivo* expansion *via* TNFRSF25 and CD25 agonists. In total, we posit future studies can take advantage of Cy based platforms to generate combinatorial strategies for long-term tolerance induction.

## Introduction

The use of cyclophosphamide administered post-transplant (PTCy) has recently provided a major advance in tolerance induction following hematopoietic stem cell transplantation (HSCT) (1, 2). Administration of 50 mg/kg Cy on days 3 and 4 post-HSCT involving HLA mismatched or HLA-matched transplants has been shown to be effective for graft vs. host disease (GVHD) prophylaxis (2–5). However, its use in Solid organ transplants (SOT) has been limited (6, 7) and reportedly did not significantly protect from immune destruction (8–10). Based on our and others previous and ongoing work in experimental HSCT), PTCy can delete or functionally suppress rapidly dividing allo-reactive (allo-rx) T cells while sparing some of the regulatory T cell (Treg) compartment, thus promoting an immune-regulatory environment (11–14). Notably, its early use to ameliorate GVHD is critically time-dependent and our studies demonstrated that deletion of rapidly (but not slowly) dividing allo-reactive T cell clones occurs following cyclophosphamide administration precisely on days 3, 4 post-HSCT (15). In the present study, we provide findings which support the notion that PTCy can be effectively employed to prolong the survival of SOTs, specifically mouse corneal allografts.

Corneal transplantation (CT) is the most common form of SOT (>180,000) performed worldwide (16, 17). Immunological rejection is the most common cause of graft failure in high risk vascularized corneal transplants and therefore advances in immune regulation to induce tolerance remains an unmet medical need as determined by the National Eye Institute (18–20). The results demonstrate that the timing of PTCy administration is critical for these CT and distinct from the kinetics employed following allogeneic HSCT. We also observed that PTCy can interfere with neovascularization, a process critically associated with immune rejection of corneal tissue that ensues following the loss of ocular immune privilege (21). Importantly, systemic PTCy treatment at doses which diminished T cell rejection responses, was observed to spare significant levels of CD4^+^ FoxP3^+^ (Tregs). Experiments also demonstrated that following such PTCy treatment, Tregs which persisted could be expanded and were functionally suppressive. In total, the present studies advance the notion that combinatory tolerance applications can be tested which involve deletional and regulatory strategies using cyclophosphamide and *in vivo* Treg expansion *via* stimulation of TNFRSF25 and CD25 receptors (22). Here, these receptors were, respectively targeted using a TL1A-Ig fusion protein and low dose IL-2 (23, 24). Through the optimization of delivery kinetics for both Cy and any applicable Treg expansion protocols, we posit this approach can be developed for application to both solid organ and liquid tissue transplants.

## Methods

### Mice

C57BL/6J (B6; stock: 000664), B6-CD45.1 breeder (stock: 002014) (H2^b^) were purchased from the Jackson laboratory and maintained in University of Miami animal facilities. The FoxP3 reporter mice on a C57BL/6 background (B6-FoxP3RFP) were originally provided by R. Flavell (Yale University, New Haven, CT). Wild-type BALB/c (H2^d^) mice and C3H (H2^k^) were purchased from Taconic or Jackson Laboratory. Mice were used at 6–12 weeks of age and were maintained in pathogen-free conditions at the University of Miami animal facilities. All animal procedures used were performed under protocols approved by the UM IACUC.

### Antibodies Used and Flow Cytometric Analysis/Phenotype

Commercial antibodies for use in flow cytometry were purchased from BD Biosciences (San Jose, CA), Biolegend (San Jose, CA), or eBioscience/ThermoFisher (Waltham, MA). Single-cell suspensions were prepared from different organs (spleen, lymph nodes [mesenteric, inguinal, axillary, and cervical]). Peripheral blood was collected in heparinized tubes. Peripheral blood mononuclear cells were isolated by standard Ficoll density gradient centrifugation. Next, 10^6^ cells were pre-blocked with anti-mouse CD16/CD32 and stained with different antibody combinations. Intracellular staining was performed according to standard procedures. The following mAbs to the indicated molecules and their fluorescent labels were used in this study: CD4, CD8, CD19, CD25, CD44, CD62L, KLRG1, CD39, CD73, I-COS, Nrp-1, PD-1, CTLA-4, Ly-6C, Ki-67, Annexin V, H2kb, H2kd, CD45.1, and CD45.2.

### Hematopoietic Stem Cell Transplantation (HSCT)

For HSCT using a major MHC-mismatch model (B6BALB/c), female BALB/c mice (H2^d^) received ablative conditioning with a single dose of 8.5 Gy total body irradiation 1 day prior to transplant. Bone marrow (BM) cells were obtained from femurs, tibias, and vertebrae from sex-matched B6-CD45.1 (H2^b^; Thy1.2) donor animals. A single-cell suspension of marrow cells was prepared by flushing bones with a 21-gauge needle and the cells were filtered through a 100-μm nylon mesh. T cell depletion (TCD) of donor marrow cells was achieved *via* complement-mediated lysis using anti-T-cell-specific antibody HO-13-4 (hybridoma supernatant, mouse anti-Thy1.2 IgM; ATCC), anti-CD4 (clone 72.4) mAb, anti-CD8 (clone H02.2) mAb (initially provided by Dr. Bruce Blazar, University of Minnesota, Minneapolis, MN), and rabbit complement (Cedarlane Laboratories, Burlington, Ontario, Canada). The marrow cells were incubated at 37°C for 45 min, washed twice in RPMI, and resuspended for hematopoietic cell transplant. Marrow TCD was routinely >99%. Donor T cells were prepared from spleens obtained from B6-FoxP3^rfp^ animals. Donor cells were stained for T cells (anti-CD4, clone RM4-5; anti-CD8, clone 53-6-7) and adjusted to 1.1 × 10^6^ T cells per mouse prior to mixing with BM. Recipient mice were transplanted (day 0) with TCD BM (5 × 10^6^) and 1.1 × 10^6^ T cells IV in a 0.2 mL volume *via* tail vein injection.

GVHD was assessed by monitoring recipients for changes in total body weight, clinical signs, and overall survival. The clinical signs of GVHD were recorded for individual mice. Recipients were scored on a scale from 0 to 2 for 6 clinical parameters modified from Cooke et al. (25): (1) weight loss; (2) diarrhea; (3) fur texture; (4) posture; (5) alopecia; and (6) mobility.

### Orthotopic Corneal Transplantation

Mice were anesthetized with ketamine (100 mg/kg) and xylazine (10 mg/kg) i.p. before the surgical procedure. Orthotopic corneal transplants were performed in non-vascularized and high-risk vascularized C57BL/6 or BALB/c mice as previously described (26, 27). Initially, a central 2-mm full-thickness trephination of the recipient cornea was performed, followed by excision with corneal scissors. Corneas from donor mice were then prepared in a similar fashion and secured to the recipient bed using eight interrupted 11-0 nylon sutures (Sharppoint, Dallas, TX). Erythromycin ointment was applied, and transplants were examined 72 h after surgery. Corneal grafts with flat anterior chamber, ulceration, or other complications related to surgical difficulties were excluded as technical failures. All corneal sutures were removed at postoperative day (POD) 7. After suture removal, corneal grafts were evaluated twice a week using slit lamp biomicroscopy and clinical scoring of clarity. A standard scoring system of 0–4 was used for corneal opacification: 0 = clear, 1 = slight haze, 2 = increased haze but anterior chamber structures are visible, 3 = advanced haze with difficult view of anterior chamber structures, and 4 = opaque cornea without view of anterior chamber structures. Grafts that received two consecutive scores ≥ 3 without resolution were considered rejected (26, 27).

### Induction of High-Risk Vascularized Corneal Recipients

Vascularization of the corneal bed in mice used as high-risk recipients was induced as previously described (28, 29). Briefly, three interrupted intrastromal 11-0 nylon sutures (Sharppoint, Dallas, TX) were placed in the central cornea of one eye of normal BALB/c mice for 14 days and these vascularized corneas were used as recipients for CT.

### Cyclophosphamide Treatment

Cyclophosphamide (MilliporeSigma, Burlington, MA) was administered i.p. (90–125 μL/injection) using varying doses between 50 and 200 mg/kg on day 3 or 3 + 4 post-HSCT (2, 15). Doses between 50 and 90 mg/kg were administered (i.p.) at the indicated times following vaccination with allogenic cells or corneal transplants.

### *In vivo* Treg Expansion Protocol

The fusion protein TL1A-Ig (50 μg) was administered i.p. on 4 consecutive days (23). Low dose IL-2 was administered as ether rmIL-2 (1.5 μg) bound to α-IL-2 mAb (clone JES6-5H4; 8 μg) or free human IL-2 (10,000 U) was injected on the final day of TL1A-Ig injection and again, 2 days later. Mice were bled or sacrificed the day following the last IL-2 injection (22).

### Alloantigen Priming and Cyclophosphamide Treatment

Alloantigen priming was performed using 10^7^ thymocytes isolated in PBS from BALB/c (H2^d^) mice injected SQ into B6-FoxP3^RFP^ (H2^b^) mice at the left lateral thoracic location. Post Vaccination Cyclophosphamide (PVCy) was given at 50 mg/kg/dose on days 3, 4, and 6 after BALB/c thymocyte SQ injections to the indicated treatment groups. Alloantigen exposed and PVCy treatment B6-FoxP3^RFP^mice were euthanized at day 21 after alloantigen vaccination. Splenocytes were harvested to establish *in vitro* mixed lymphocyte reaction (MLR) assays. Responders (CD4^+^ and CD8^+^ lymphocytes) from the alloantigen and PVCy B6-FoxP3^RFP^ treatment groups were plated in 96 well flat-bottom plates at 1 × 10^5^ cell/well in triplicate cultured in RPMI media with 2% Fetal Bovine Serum. Responders were co-cultured in the presence of stimulator antigen presenting cells (APC's) irradiated with (20 Gray) from BALB/c (H2^d^) or C3H (H2^k^) splenocytes at 2 × 10^5^ cells/well. Cultures were incubated for 60 or 132 h. and pulsed with [3H]-thymidine (0.5 Ci/well) for 12 h. Responders were harvested at days 3 and 6 for CPM counts of incorporated [3H]-thymidine isotope measured by liquid scintillation counting (Micro Beta TriLux Counter, Perkin Elmer, Waltham, MA) (30).

### Mixed Lymphocyte Response

Mixed lymphocyte responses (MLR) were performed using PMCS (2 × 10^5^) from BALB/c CT recipients or splenocytes from B6 animals were used as responders and were stimulated with 1 – 2 × 10^5^ irradiated B6 (H2^b^, donor), BALB/c (H2^d^, host), or C3H/HeJ (H2^k^, third party) splenocytes. Responders and stimulators were co-cultured in 96-well round-bottom plates for 120 h and pulsed with [^3^H]-thymidine (0.5 μCi/well; Perkin Elmer, Waltham, MA) for the final 16 h. Incorporated isotope was measured by liquid scintillation counting (Micro Beta TriLux counter; Perkin Elmer) and results presented as mean cpm/group.

### Statistical Analyses

All graphing and statistical analysis were performed using GraphPad Prism (San Diego, CA). Values shown in graphs represent the mean of each group ± SEM. Survival data were analyzed with the Mantel-Cox log-rank test or Gehan–Breslow–Wilcoxon. Non-parametric unpaired two-tailed *t*-test was used for comparisons between two experimental groups, and multiple variable analysis was performed using 2-way ANOVA. A *p* < 0.05 was considered significant with Bonferroni or Turkey correction for repeated measures of multiple comparisons. Brackets (or as described in the legends) identifying the groups being compared are presented in each figure where appropriate accompanied by the level of significance or absence of significance (ns).

## Results

### Cyclophosphamide Treatment Post-hematopoietic Stem Cell Transplant Ameliorates GVHD or Post-corneal Transplant Can Delay Allograft Rejection Times

**Figure 1:**
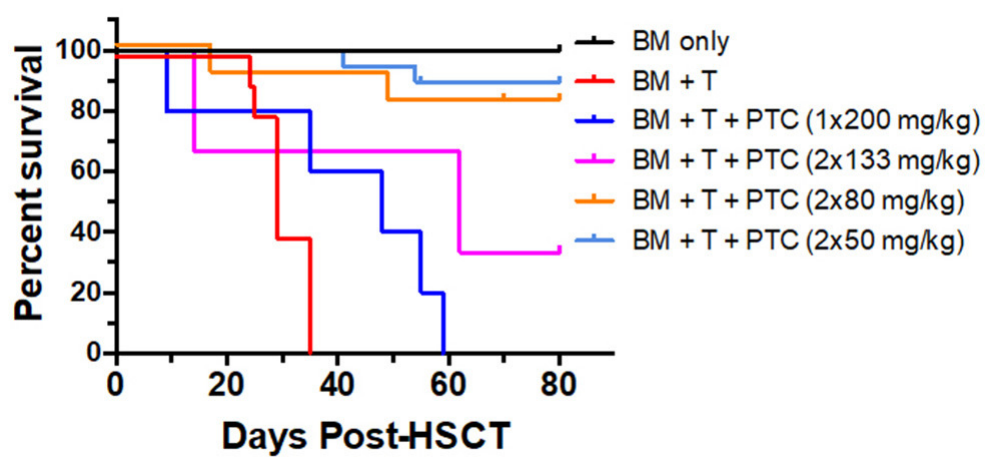
PTCy treatment early post-transplant ameliorates GVHD long-term/delays allograft rejection in a major MHC mismatch model of pre-clinical HSCT. A HSCT utilizing a B6 BALB/c donor/recipient mouse model involving a complete MHC mismatch was performed on day 0. Lethally irradiated (8.5 Gy) BALB/c mice received 5 × 10^6^ TCD B6-CD45.1 BM cells alone or with spleen cells from B6-FoxP3^RFP^ donor mice adjusted to contain 1.1 × 10^6^ total T cells. Cyclophosphamide was given on day 3, 1 × 200 mg/kg or day 3 and 4 post-HSCT at 133, 80 and 50 mg/kg i.p., respectively. Survival of the different groups is shown (BM *n* = 5; BM + T *n* = 10; BM + T + 1 × 200 mg/kg *n* = 5; BM + T + 2 × 133 mg/kg *n* = 3; BM + T + 2 × 80 mg/kg *n* = 9; BM + T + 2 × 50 mg/kg *n* = 14). BM vs. 1 × 200 *p* = **; BM vs. 2 × 133 *p* = *; BM vs. 2 × 80 *p* = ns; BM vs. 2 × 50 *p* = ns; BM + T + No PTCy vs.: 1 × 200 *p* = *; 2 × 133 *p* = ns; 2 × 80 *p* = ***; 2 × 50 *p* = ****; 1 × 200 vs. 2 × 133 *p* = ns; 1 × 200 vs. 2 × 80 *p* = **; 1 × 200 vs. 2 × 50 *p* = ****; 2 × 133 vs. 2 × 80 *p* = ns; 2 × 133 vs. 2 × 50 *p* = ns; 2 × 80 vs. 2 × 50 *p* = ns. *p < 0.05; **p < 0.01; ***p <0.001; ****p <0.0001. Data for these transplants represents 2 pooled independent experiments for all groups except BM only and BM + T + 2 × 133 mg/kg.

Our work and others have shown that administration of post-transplant cyclophosphamide (PTCy) can improve outcomes in MHC-mismatched pre-clinical and clinical aHSCT (31–33). To more precisely address the dose of PTCy which can ameliorate pre-clinical GVHD in this model, transplants were performed across a complete MHC disparity and varying doses of PTCy were administered on days 3 + 4 to BALB/c (H2^d^) recipients following transplant with B6 (H2^b^) donor cells (Figure 1). Reduced GVHD clinical scores were observed at each treatment dose assessed. The single D.3 application of 200 mg/kg resulted in the lowest improvement of survival time. Days 3 and 4 (D3 + 4) dosing of 50 or 80 mg/kg clearly reduced mortality (0 % survival in untreated group vs. 80–90% survival in PTCy 50–80 mg/kg treated). These findings demonstrate that D3 + 4 delivery of Cy following donor bone marrow plus T cell replete allografts can markedly diminish GVHD and improve HSCT outcomes.

**Figure 2:**
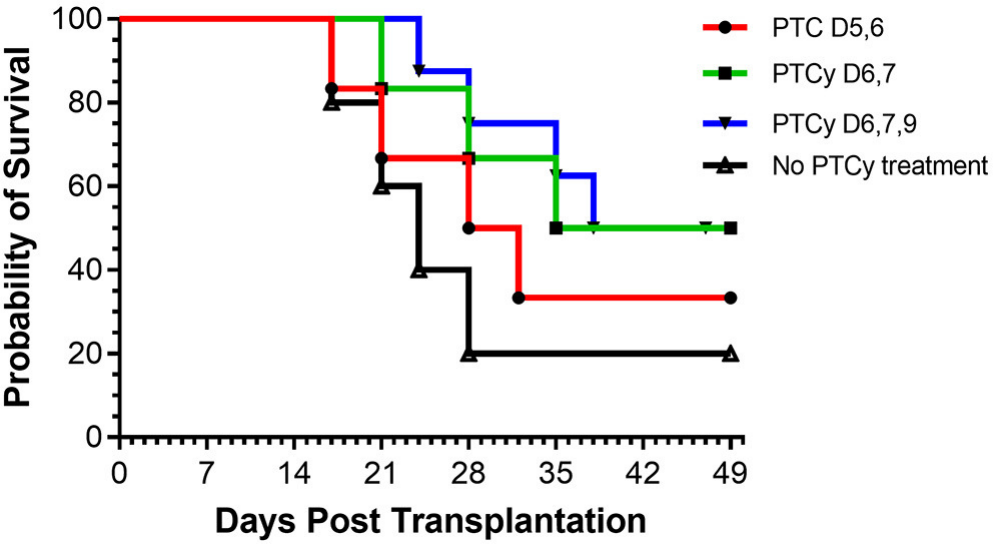
Kinetics of PTCy treatment following MHC mismatched corneal allografts (B6 BALB/c). Groups of BALB/c recipients received MHC-mismatched B6 corneal grafts and on either days 5, 6 or 6, 7, or 6, 7, 9 were i.p. injected with cyclophosphamide (70 mg/kg). PTC D5, 6 (*n* = 6) vs. Control (*n* = 5) *p* = ns; PTC D6, 7 with or without PTC D9 (*n* = 14) vs. Control (*n* = 5) *p* = *. *p <0.05.

**Figure 3:**
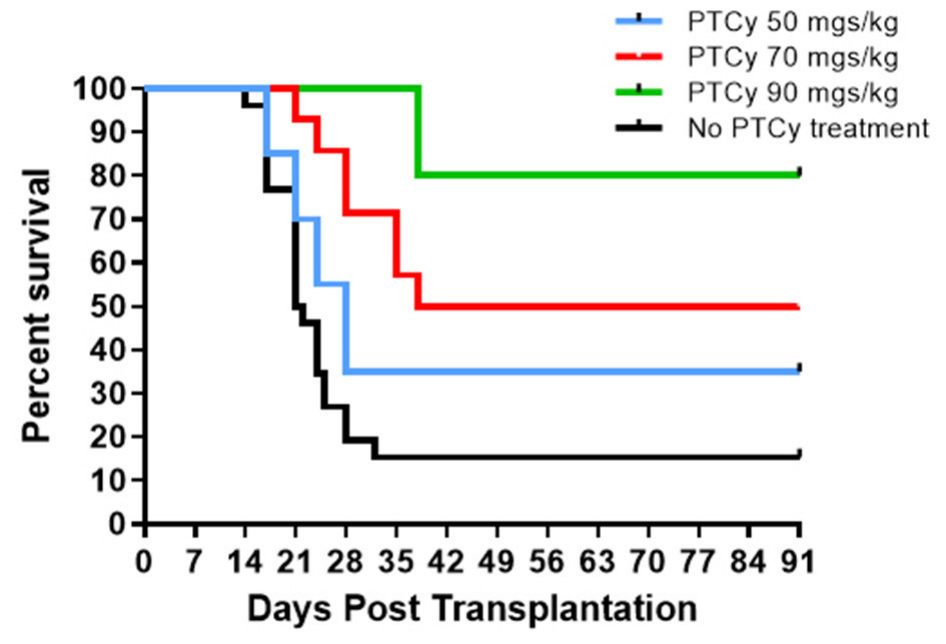
Post-orthotopic corneal allograft treatment with PTCy significantly delays graft rejection. BALB/c mice received MHC-mismatched B6 corneal grafts and groups did not receive PTCy treatment (*n* = 26, 5 pooled independent experiments) or on days 6 and 7 received either 50 (*N* = 20, 4 pooled independent experiments), 70 (*N* = 14, 3 pooled independent experiments) or 90 (*N* = 5) mg/kg cyclophosphamide i.p. No PTCy treatment vs. 50 *p* = ns; No PTCy treatment vs. 70 *p* = ***; No PTCy treatment vs. 90 *p* = **. **p <0.01; ***p <0.001.

Based on these findings, we asked if use of cyclophosphamide post-transplant may prolong survival of solid tissue allografts. We are interested in corneal transplants (CT) because of the high annual numbers performed clinically and the ready accessibility of the ocular compartment lends itself for local delivery of reagents and therefore translational application. Complete MHC-mismatched CT were performed using B6 (H2^b^) donor tissue and BALB/c (H2^d^) recipients. Because corneal transplants, specifically those that have non-vascularized recipient beds or low risk CT, relative to other SOTs lack the ability to elicit rapid rejection through the direct antigen presentation pathway (34), we anticipated that D3 + 4 PTCy may not be optimally effective. Reportedly the cervical lymph nodes (CLN) are a draining tissue for corneal allografts (35) and examination of CLN at days 3, 4, 5, 6 found a transient decrease in CD4^+^ and CD8^+^ T cells on day 4 post-Cy injection and then immediate return to normal levels (Supplementary Figure 1). We reasoned that application of cyclophosphamide beyond days 3 and 4 may be more effective to regulate corneal graft rejection so Cy was administered at different time points post-CT (Figure 2). Compared to untreated (“control”) recipients, treatment with 70 mg/kg at D6, 7 ± 9 significantly prolonged CT whereas earlier PTCy, i.e., at D.5 + 6 did not. Next, transplant experiments compared the administration of 70 and 90 −50 mg/kg, the latter which effectively inhibited B6BALB/c hematopoietic stem cell grafts (Figure 1) (31). Results from BALB/c recipients receiving 70 mg/kg on D6 + 7 post-transplant of B6 CT allografts indicated that this dose was superior to 50 mg/kg for prolonging these CT allografts and 90 mg/kg was found to have the most pronounced effect on prolonging graft survival (Figure 3). Additionally, Cy treatment at D3 + 4, despite a 70 mg/kg dosage failed to prolong graft survival in high risk vascularized CT (Supplementary Figure 2).

### PTCy Treatment Following Corneal Allografts Also Reduces Neovascularization and Diminishes Anti-donor Alloantigen MLR Responses

**Figure 4:**
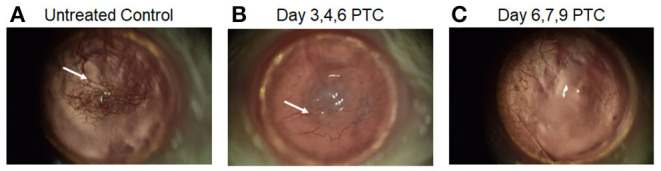
Slit-Lamp images of post-transplant cyclophosphamide effect on neovascularization day 60 post high-risk corneal transplant. BALB/c mice were vascularized (using 3 corneal sutures) 14 days prior to receiving a B6 corneal allograft. **(A)** untreated recipients, **(B)** PTCy treated on days 3, 4 and 6 post-CT with 75 mg/kg; **(C)** PTCy treated on days 6, 7, and 9 post-CT with 75 mg/kg. Newly formed blood vasculature appears as red vessels (white arrows) in the central cornea.

Vascularization of the corneal bed accompanies and is required for immune rejection of these allografts. Neovascularization of corneas were examined approximately 2 months post-transplant in untreated and PTCy treated allograft recipients to validate that acceptors vs. rejectors were being assessed. High risk vascularized corneal beds were induced by intrastromal sutures applied 2 weeks prior to transplant (Supplementary Figure 3). B6 corneal tissue was transplanted onto these BALB/c recipients followed by earlier or later PTCy treatment. Slit lamp examination of grafted corneas from recipients of cyclophosphamide administered on days 6, 7 and 9 (D6, 7 + 9) exhibited the fewest vessels in the central corneal region (Figure 4). Thus, the treatment timing of PTCy was found to significantly prolong allograft survival and was associated with substantially diminished neovascularization compared to untreated control recipients.

**Figure 5:**
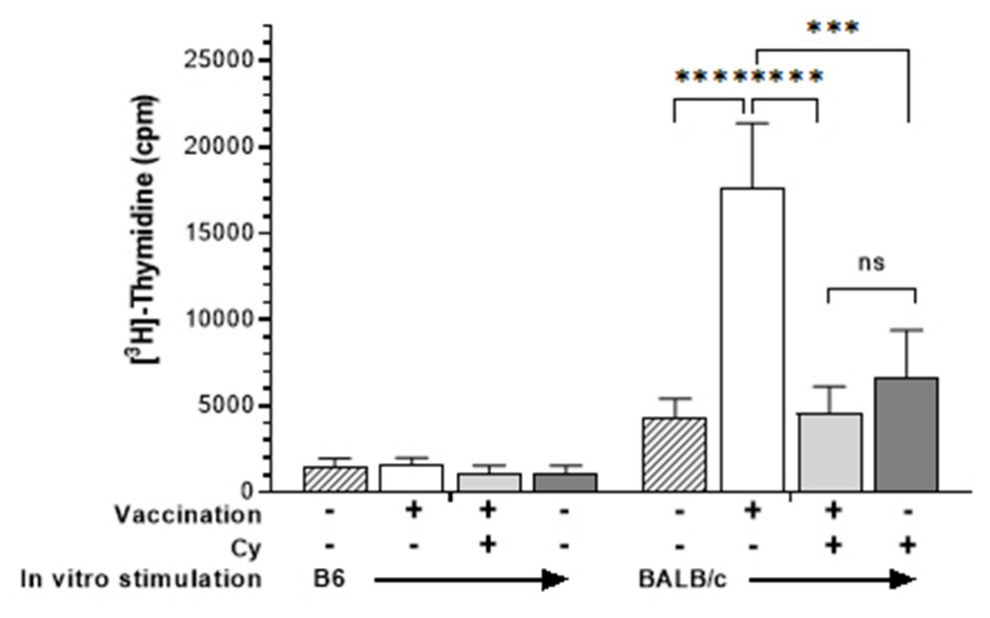
Cyclophosphamide treatment reduces recipient T cell response to alloantigen. Spleen cells were harvested from B6 animals who were unimmunized or received allogeneic vaccination with BALB/c spleen + thymocytes (see Methods). Some groups also received cyclophosphamide (50 mg/kg) 3, 4, and 6 days following immunization. Spleen cells were obtained 21 days after vaccination and cultured for 120 h with irradiated B6 (syngeneic) or BALB/c (allogeneic) spleen cells. Cells were pulsed with ^3^[H]-Thymidine for the final 18 h and results are presented as mean cpm/6 well replicates. Groups were compared using two-way ANOVA: ns, not significant; ***p <0.001; ****p <0.0001.

Since neovascularization is directly associated with CT rejection (36, 37) and neovascularization can be promoted by anti-graft alloreactive T cells (26) we reasoned that Cy treatment resulted in diminishing these anti-donor alloantigen specific T cell responses (13). To initially investigate the effect of cyclophosphamide administration on host alloantigen responses, mice were vaccinated with an MHC-mismatched cell inoculum. Groups of B6 mice unvaccinated or vaccinated against BALB/c alloantigen (splenocytes + thymocytes), received vehicle or 50 mg/kg Cy (D3, 4 + 6). Three weeks after BALB/c inoculation, MLRs were performed using spleen cells from vaccinated or unvaccinated mice (Figure 5). Results demonstrated that, after alloantigen exposure (vaccinated mice) as anticipated, spleen cells from non-Cy treated mice demonstrated a strong MLR response to the specific immunizing antigen. In contrast, mice receiving post-vaccination Cy (PVCy) had a diminished MLR response after stimulation with the specific immunizing antigen (Figure 5).

**Figure 6:**
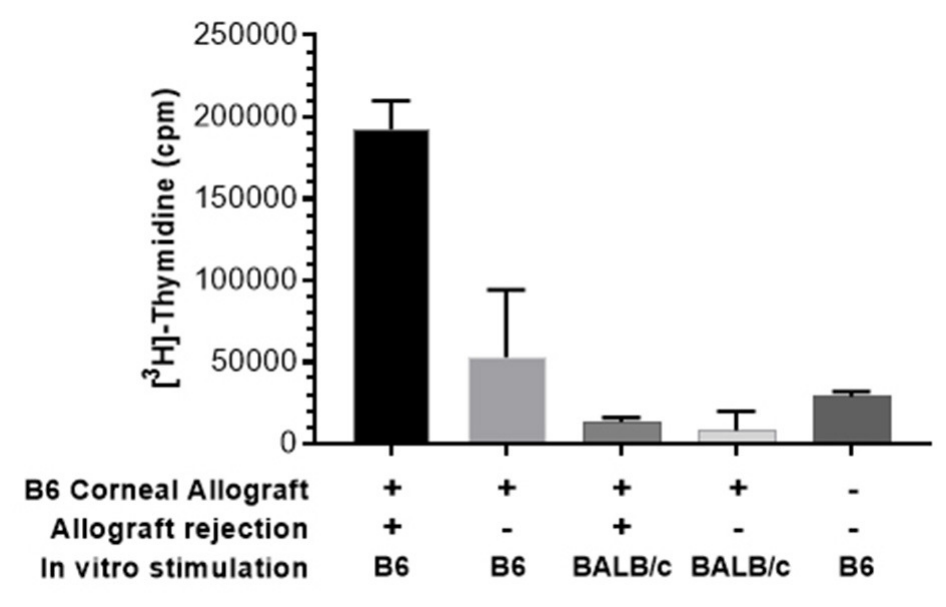
MLR using pooled peripheral blood (PB) of recipients after PTCy demonstrates decreased response against donor antigen by CT acceptors but not rejectors. PB was obtained from BALB/c recipients of B6 corneal allografts and treated with PTCy (70 mg/kg) on days 6 and 7 (as described Figure 2) or un-transplanted BALB/c. Following ficoll hypaque separation, PBMC were harvested and stimulated with irradiated B6 (donor) or BALB/c (self) spleen cells. MLR cultures were pulsed with ^3^[H]-Thymidine for the final 18 h. and assessed following 5 days in culture. Data obtained using pooled PB from each group: *n* = 2–3\g. Data is presented as the mean cpm from triplicate microwell cultures. Data represents an individual experiment.

The effect of PTCy on host T cell responses against alloantigen following a solid tissue transplant was then investigated. PTCy (70 mg/kg) was administered on D6 + 7 to BALB/c recipients of MHC-mismatched B6 corneas. Approximately 2 months following transplant, PBMCs were obtained from recipients and co-cultured together with donor (H2^b^) or autologous (H2^d^) stimulating cells. Similar to the vaccinated mice above, untreated transplant recipients who had rejected their grafts responded strongly to stimulation with donor but not “autologous” (self) antigen (Figure 6). In contrast, PTCy treated recipients who had maintained their grafts (acceptors) generated significantly lower responses following stimulation with donor antigen (Figure 6).

### PTCy Treatment That Effectively Inhibits Allo-Antigen Responses Arising After Liquid and Solid Tissue Grafts Does Not Ablate—and Allows for Expansion of the Treg Compartment

**Figure 7:**
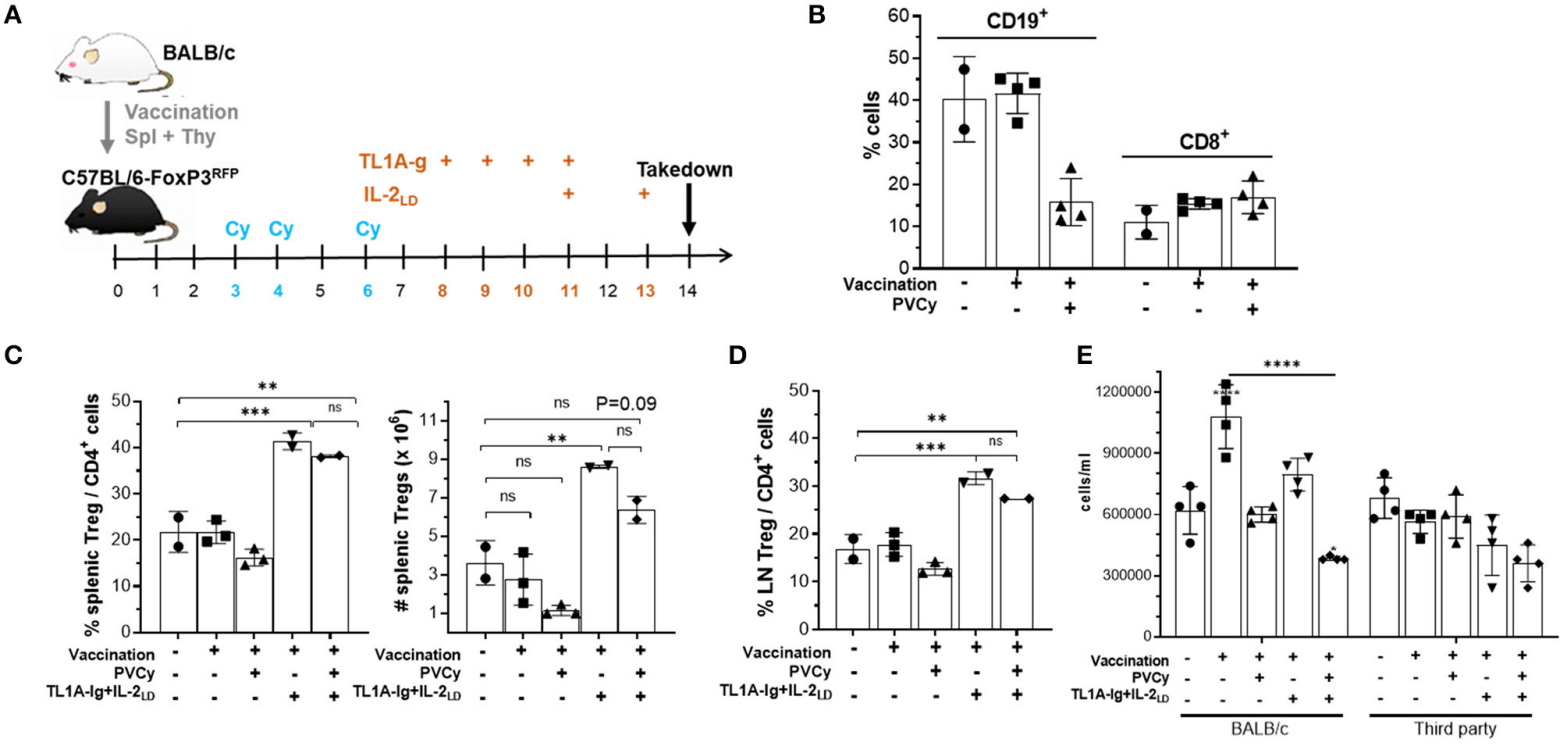
The effect of cyclophosphamide treatment on the Treg compartment: persistence and function. **(A)** Experimental design of the allogenic immunization model used in these studies. B6-FoxP3^RFP^ reporter mice were immunized with allogeneic BALB/c 10 × 10^6^ splenocytes and thymocytes. Following immunization, some groups also received cyclophosphamide (50 mg/kg = PVCy) on days 3, 4, and 6 and/or TL1A-Ig + IL-2_LD_ on days 8–13 as indicated in the figure. **(B)** Mice were bled on day 7 and frequency of CD19 ^+^ and CD8 ^+^ cells was determined (*n* = 2–4 mice/gr.). **(C–E)** On day 14 mice were sacrificed and assessed. **(C)** Splenic overall Treg frequency (%) within the CD4 fraction (CD4^+^ FoxP3^+^/CD4 ^+^) cells (*left*) and total numbers splenic Tregs (*right*) are shown (*n* = 2–3 mice/gr.). **(D)** Treg (CD4^+^ FoxP3^+^) frequency (%) of total CD4^+^ cells in LN (*n* = 2–3 mice/gr.). **(E)** Spleen cells were cultured for 120 h with irradiated BALB/c (allogeneic) or C3H (third party) spleen cells. The number of cells/mL (all groups were plated in total volume of 0.2 mL) was determined for each group. Results are presented as mean cpm/4 well replicates. Groups were compared using one-way ANOVA with Tukey's multiple comparisons test for multiple groups. **p <0.01; ***p <0.001; ****p <0.0001. Data represents 1 of 2 independent experiments.

To examine the Treg compartment after Cy exposure mice were administered alloantigen and then cyclophosphamide. Mice were vaccinated with complete MHC disparate allogeneic spleen + thymocytes, administered 50 mg/kg cyclophosphamide D3, 4 + 6 days later and then assessed for Treg presence and phenotype (Figure 7A). As anticipated, animals treated with PVCy exhibited a significant loss of B cells (CD19^+^) but not CD8^+^ T cells (Figure 7B). Two days following the last injection of cyclophosphamide (D6), some animals received agonistic reagents which target TNFRSF25 (TL1A-Ig fusion protein) and CD25 (IL-2_LD_) receptors that can selectively stimulate rapid Treg expansion (22). Following a 6-day TL1A-Ig + IL-2_LD_ (“2-pathway”) treatment protocol, Treg expansion was assessed. In contrast to mice not receiving this treatment, animals receiving the 2-pathway expansion protocol exhibited significantly elevated CD4^+^ FoxP3^+^/CD4^+^ lymph node and splenic Treg levels, 15–25% vs. 38–43%, respectively (Figures 7C,D). Notably much higher numbers of Tregs were also induced post-expansion with or without Cy treatment (Figure 7C). Treg central and effector subset distribution, analyzed by CD62-L and Ly-6C expression (38, 39), and KLRG1 expression marking terminal differentiation were found unaltered in vaccinated mice who did and did not receive PVCy treatment (Supplementary Figures 4A,B). We also examined vaccinated mice receiving the Treg expansion protocol. Mice who did or did not receive Cy treatment, similarly exhibited no differences in Treg subset distribution (Supplementary Figures 4A,B).

Following Cy treatment, Tregs obtained from vaccinated animals receiving the TL1A-Ig + IL-2_LD_ expansion protocol exhibited suppressor activity comparable to animals not receiving Cy treatment (Supplementary Figure 4C). Notably, Cy treatment following priming reduced the MLR response by spleen cells against the priming antigen but not against a third-party alloantigen (Figure 7E). Treg expansion following Cy treatment further reduced the inhibition observed by Cy alone (Figure 7E). In total, these experiments demonstrated that using doses of cyclophosphamide that diminished responses against alloantigen resulted in: (a) some Tregs persisting after PTCy treatment and (b) persisting Tregs capable of undergoing marked expansion following stimulation *via* TNFRSF25 and CD25 stimulation.

**Figure 8:**
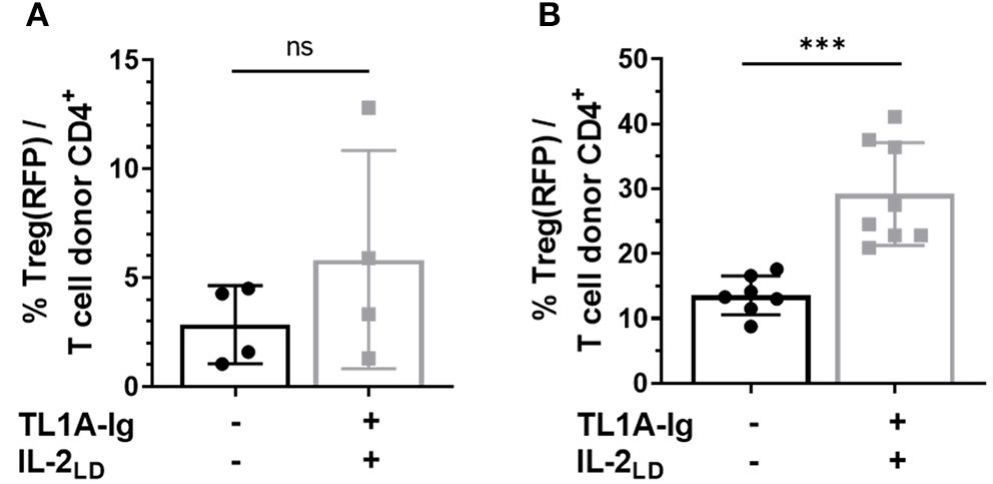
Tregs survive cyclophosphamide treatment post-HSCT and can be expanded. HSCT utilizing a B6 BALB/c complete MHC mismatch was performed on day 0. Lethally irradiated (8.5 Gy) BALB/c mice received 5 × 10^6^ TCD B6-CD45.1 BM cells and spleen cells from B6-FoxP3^RFP^ donor mice adjusted to contain 1.1 × 10^6^ total T cells. Cyclophosphamide was administered i.p. on days 3 and 4 post-HSCT **(A)** at 80 mg/kg and **(B)** 50 mg/kg. **(A)** Treg expansion was initiated on day 2 post PTCy (D.6 post-HSCT) by giving 50 μg TL1A-Ig for 4 consecutive days and IL-2 (complex of 1.5 μg rmIL-2 bound to 8 μg α-IL-2 mAb (clone JES6-5H4) on the final day of TL1A-Ig and 2 days later. Donor Treg expansion analyzed by flow cytometry 1 day after the last IL-2 dose does not reach significance (*p* = ns). **(B)** Treg expansion was initiated on day 50 post PTCy (D.54 post-HSCT) by giving 50 μg TL1A-Ig on 4 consecutive days and IL-2 (free, hu10,000 U) on the final day of TL1A-Ig and 2 days later. Donor Treg frequency analyzed by flow cytometry 1 day after the last IL-2 dose is presented as the % CD4^+^ FoxP3^+^/total CD4^+^ T cells in recipient peripheral blood. ns = *p* > 0.05; ***p <0.001. Data represents 2–3 pooled independent experiments.

Treg presence following PTCy for aHSCT was required for optimal amelioration of preclinical GVHD (11–13). We examined the Treg compartment following doses of PTCy found effective to inhibit GVHD following aHSCT for: (a) CD4^+^ FoxP3^+^ cell presence and (b) Treg function. Experiments analyzed the homeostatic Treg compartment post-HSCT and after administration of TL1A-Ig + IL2_LD_ as above. In these experiments, recipients received 8.5 Gy TBI conditioning followed by PTCy (50 or 80 mg/kg) on D3 + 4. Transplanted donor CD4^+^ FoxP3^+^ Tregs were detected, i.e., 2–3% and 10–15% of CD4 ^+^ donor cells in recipient blood 1 and 7 weeks following PTCy, respectively (Figure 8). Administration of TL1A-Ig fusion protein and IL-2_LD_ initiated 2 days after PTCy (i.e., on D6) or 50 days after PTCy (D54) resulted in minimal expansion of the surviving transplanted splenic donor Tregs at 1 week. but significant expansion at 8 weeks, respectively (Figure 8). These findings indicated that some populations of donor Tregs persist following PTCy in TBI conditioned aHSCT recipients and both the levels of Tregs following PTCy and their subsequent ability to be expanded were dependent on the time following conditioning and HSCT.

**Figure 9:**
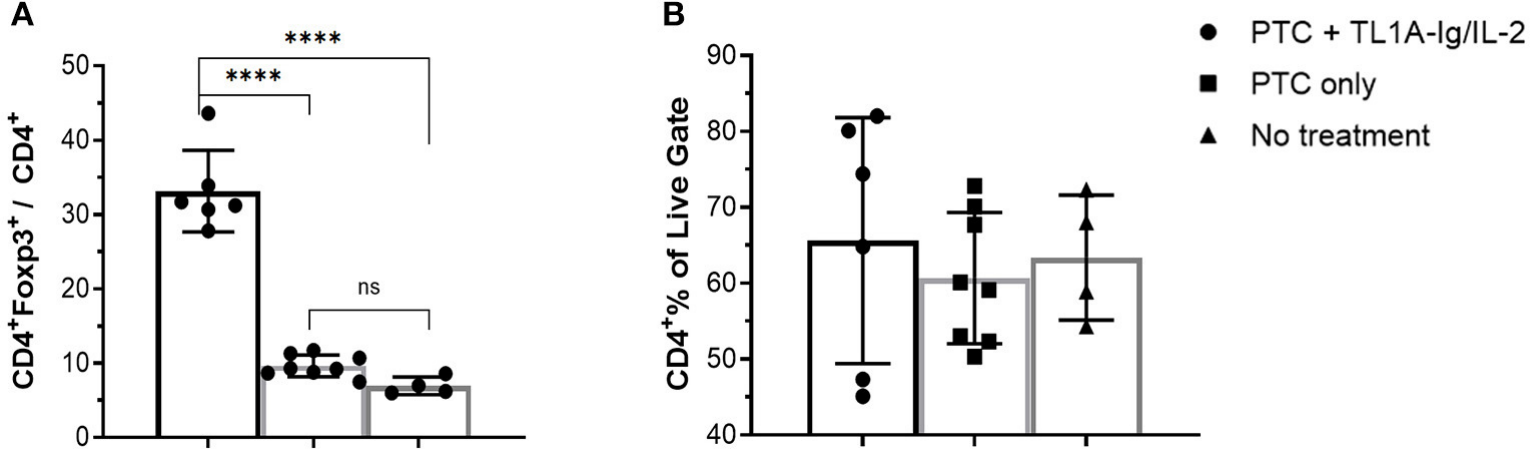
TL1A-Ig + IL-2 induces significant Treg expansion in blood following post-orthotopic corneal transplant administration of PTCy. Recipient B6-FoxP3^RFP^11 mice were transplanted with BALB/c corneal grafts and injected with TL1A-Ig + IL-2_LD_ beginning D10 post-CT (see Methods). PTCy was administered i.p. on D6, 7 + 9. Mice were bled on D16 and PBMC were stained to assess FoxP3 **(A)** and CD4 **(B)** expression by flow cytometry: ANOVA: ns = *p* > 0.05; ****p <0.0001.

Regulatory T cells also play a critical role in regulating responses to SOTs (9, 40, 41). Treg presence following CT and PTCy treatment would provide opportunity to manipulate these cells as an approach to augment tolerance to graft antigens. A key question to address is whether activation of the co-stimulatory TNFRSF25 molecule to expand Tregs may unwantedly drive anti-graft effectors accelerating graft rejection. Mice were therefore transplanted with MHC-mismatched corneal tissue and groups were treated with 70 mg/kg Cy on D6, 7 + 9. One day later, a group received TL1A-Ig and IL-2_LD_ over 6 days as described above. CT recipients who did not or did receive PTCy contained 7–10% Tregs within their PB CD4^+^ T cells ~ 2 weeks post-CT (Figure 9A). Notably, TNFRSF25 and CD25 stimulation significantly expanded the Tregs present in these PTCy treated animals but did not significantly alter the overall CD4 compartment (Figure 9). These findings demonstrate that some Tregs also clearly persisted after solid tissue grafting and the Cy treatment administered and expanded following the 2-pathway stimulation protocol.

### Combining Use of PTCy Together With Expansion of Tregs Following Corneal Transplant

**Figure 10:**
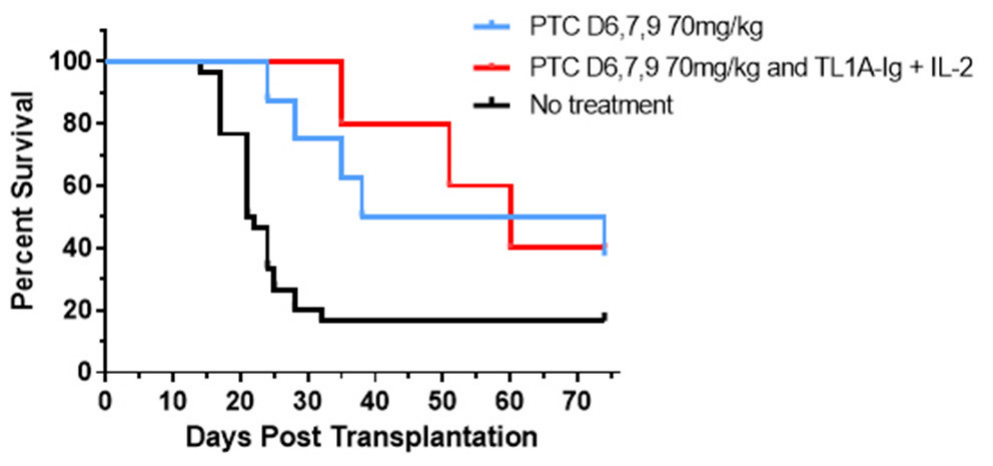
Post orthotopic corneal allograft treatment with PTCy and TL1A-Ig + IL-2 delays graft rejection. BALB/c mice transplanted with B6 corneal grafts. Mice were untreated (*n* = 30, 5 pooled independent experiments) or PTCy only (70 mg/kg) on days 6, 7 + 9 (*n* = 8, 3 pooled independent experiments) or PTCy followed by TL1A-Ig + IL-2_LD_ beginning D.10 post-CT (*n* = 5). PTC D6, 7 + 9 vs. No treatment *p* = **; PTC D6, 7 + 9 and Treg vs. No treatment *p* = *; PTC D6, 7 + 9 vs. PTC D6, 7 + 9 and Treg *p* = ns. *p <0.05; **p <0.01.

To begin addressing the potential application of a combinatorial tolerance strategy, B6 corneal grafts were placed on groups of BALB/c animals and some were administered 70 ms/kg PTCy on D6, 7 + 9. These PTCy recipients (Figure 10) again demonstrated a significant increase in graft survival time vs. untreated animals (see Figure 2). PTCy treated CT recipients who subsequently received our brief 6-day TL1A-Ig + IL-2_LD_ treatment protocol initiated immediately following PTCy (days 10–15) also significantly differed from untreated recipients and demonstrated no significant decrease in graft survival vs. CT recipients treated with PTCy alone (Figure 10). In summary, these findings do not support the notion that TNFRSF25 stimulation after PTCy drives anti-graft effector cells accelerating graft rejection but alternatively, this strategy can be applied to expand as well as maintain Tregs following Cy treatment to further suppress immune mediated graft rejection.

## Discussion

PTCy has been found to be an effective graft vs. host disease prophylactic treatment strategy following pre-clinical and clinical HSCT (1, 2, 5, 13). The present study investigated if application of cyclophosphamide following a pre-clinical solid tissue transplant, i.e., corneal allografts could delay host vs. graft rejection and prolong survival. Prior studies using cyclophosphamide following CT did not observe prolonged allograft survival (8, 9). In contrast, the findings here demonstrated that PTCy usage can prolong survival of these CT when administered at selective times distinct from optimal Cy kinetics post-HSCT (1, 31, 42). Results also identified the presence of Treg cells following PTCy treatment in both the pre-clinical liquid and solid tissue graft models. Additionally, these persisting Tregs could be expanded at different time periods following Cy treatment providing opportunity to implement combinatorial strategies to augment immune tolerance toward permanent graft acceptance.

Administration of cyclophosphamide for other solid tissue graft tolerance has been previously considered and was reported to prolong murine allogeneic skin grafts (6, 7, 43, 44). The application of PTCy following liquid and SOT must take into account several differences between the two procedures. First, for successful solid organ/tissue transplant outcomes, vascularization must occur delivering local oxygen and nutrients to the allograft. Results here found that unlike HSCT where GVHD can be suppressed, administration of PTCy on D3 + 4 post-transplant of corneal tissue was ineffective at prolonging graft survival. Examination of D3, 4 treated CT grafts several days later demonstrated poor wound closure (data not shown) suggesting PTCy administered too early post-keratoplasty may have negative or deleterious impact on ocular parenchymal tissue (45) as well as inadequate immune suppression of alloreactive T cells. It should be noted that corneal neovascularization and lymphangiogenesis leads to loss of immune privilege in the anterior compartment thereby contributing to graft rejection (21, 26, 37, 46). We detected diminished corneal neovascularization in recipients following later cyclophosphamide administration, i.e., day 6, 7, and 9 (75 mg/kg) which was associated with extended graft survival. Thus, the timing of PTCy is likely critical regarding angiogenic inhibition. Cyclophosphamide has been found to reduce neovascularization in the absence of alloreactive T cells (47). Additionally, PTCy deletes/suppresses effector T cell populations which likely include those subsets found to produce angiogenic factors driving neovascularization. Thus, PTCy administration at this time (D6, 7 + 9) post-grafting may provide additional benefit through the ability to inhibit neovascularization by either direct inhibition of vascular endothelial cells and/or *via* immune mediated signals driving angiogenesis and lymphangiogenesis. Together such mechanisms could contribute to extending the survival of CT allografts.

The kinetics of the immune responses leading to graft rejection also likely differ following liquid bone marrow and solid corneal tissue transplants. Following T cell replete HSCT, donor T cells almost immediately encounter host APC in lymphoid compartments initiating activation leading to proliferation and elicitation of anti-recipient effector cell responses (48). Concerning solid organ allografts, non-vascularized corneal tissue grafts compared to skin grafts possessing high levels of APC/DC, involve slow lymphoid and vascular drainage delaying antigen presentation following these transplants. These low risk CT relative to other SOT also lack the ability to elicit rejection through direct allo-responses by CD4^+^ T cells and therefore reject through the slower indirect pathway (34). Thus, it is not surprising that the timing of administration of effective Cy treatment to prolong these CT grafts compared to HSCT was not identical, i.e., D6, 7 + 9 for the former and D3 + 4 for the latter. This delayed treatment schedule was fortuitously beneficial also avoiding as noted above, direct corneal graft damage early post-surgical implant. PTCy administration on days 6 and 7 following CT which prolonged allograft survival also diminished *in vitro* anti-donor alloantigen T cell responses in mice which accepted their grafts, in contrast to non-Cy treated graft rejectors.

Treatment with cyclophosphamide is anticipated to damage rapidly dividing lymphoid cells as a consequence of DNA alkylation and recent studies also report that Tregs persisting following PTCy treatment possess suppressive function important in overall immune regulation following liquid HSCT (13). Tregs have been found to augment survival of solid organ allografts including CT (49–51). Combining T cell deletion and immune regulation involving the manipulation of Tregs following cyclophosphamide treatment may represent an advance toward inducing and maintaining immune tolerance. A number of studies have reported cyclophosphamide effects on Tregs which are dependent on the dosing and timing of drug delivery (52, 53). Some earlier reports discussed the use of cyclophosphamide to eliminate Tregs and augment cancer vaccine effectiveness in antitumor immune responses (54). However, our and others prior reports demonstrated that significant levels of Tregs can persist after Cy administration dependent on dose and duration following TBI conditioning and hematopoietic stem cell transplantation (11, 12, 42). These findings are consistent with reports showing some Tregs are resistant to genomic insults which include TBI (55–57). Moreover, these Tregs were required for optimal PTCy protection from GVHD post-HSCT indicating some functionality was retained (12). Elevated levels of ALDH are present in Tregs and promote catabolism of the drug preventing generation of the alkylating mustard compound (11). Together with the doses used (<200 mg/kg total) here and brief exposure ≤ 3 injections), Tregs clearly persisted after Cy administration following vaccination with alloantigen and application of both solid (CT) and liquid (HSCT) allogeneic transplants. Results in the present study showed that CD4^+^ FoxP3^+^ Tregs can be expanded in CT and vaccinated recipients almost immediately following cessation of PTCy treatment through stimulation with TNFRSF25 and CD25 receptor agonists. This is an important finding as we have observed immediately following TBI and allo HSCT, Treg expansion cannot be similarly accomplished (DW, RBL unpublished observations). Such observations suggest that the microenvironment necessary for Tregs to expand in response to appropriate receptor stimulation is not obviated by certain Cy treatment schedules. In total, these findings suggest that strategies to combine Cy treatment with Treg expansion can be developed to strengthen tolerance protocols. We recently began to develop such protocols using multiple injections of a new agonistic anti-TNFRSF25 mAb (generated by a biotech company) together with Cy following corneal transplants that shows significant promise (unpublished data, Liwen Lin, CL,VLP, RBL).

A potential complication to such approaches could occur if effector cells were unintentionally co-stimulated by our Treg expansion protocol driving effector cell expansion and function resulting in rapid rejection or accelerated GVHD. TNFRSF25 has been shown to be expressed on activated T cell populations (58). Early application of an agonistic TNFRSF25 mAb alone have been found to result in enhanced GVHD when administered peri-transplant (59). Importantly, our protocol of TL1A-Ig fusion protein + rIL-2 utilized low dose IL-2 which has been shown to selectively stimulate Treg cells vs. conventional CD4 and CD8 T cells (60–62). Notably, we did not identify a heightened rejection response against CT following PTCy administration. We posit this may be a result of the deletion and/or Treg mediated suppressive environment regulating anti-graft effector cells which may have persisted. While not statistically significant, slower rejection may have occurred following PTCy treatment together with the immediate and one-time Treg expansion treatment compared to PTCy treatment alone. Our overall objective is to develop protocols which combine PTCy treatment and TNFRSF25 stimulation together with ultra-low dose IL-2 to optimize and extend the kinetics of Treg cell presence and function to prevent activation of any residual or future anti donor graft effector cells. This could be approached by employing prolonged Treg expansion regimens.

Overall, the findings presented here show that PTCy can be effective in prolonging both liquid and solid tissue allografts. Our data supports the notion that the timing of treatment and concentration of Cy is crucial for both the protection of solid organ grafts from direct drug damage as well as effective suppression of anti-graft T cell responses during the period when they are sensitive to regulation. The studies also demonstrated that some Tregs persist after CT following Cy treatment dependent on the dose and importantly, can readily receive stimulating signals leading to their expansion. The observation that Tregs can prolong the survival time of these allografts together with PTCy treatment is consistent with a recent report that sustained (i.e., 6 weeks post-CT) systemic administered IL-2 treatment alone could prolong CT allografts (50). In the present studies, only short-term expansion of Tregs was interrogated using 3 injections which concluded only 15 days post-CT. As previously mentioned, our data using both a TL1A fusion protein (TNFRSF25) and IL-2_LD_ (CD25) stimulation, do not demonstrate aggravation of anti-graft effector cells that may have survived PTCy treatment. Therefore, we propose studies extending the period of Treg expansion by providing continued low dose IL-2 treatment or intermittent application with TNFRSF25 + CD25 agonists. We are interested in investigating if local administration of TNFRSF25/CD25 agonists, specifically within the ocular adnexa may be particularly effective at targeting and limiting Treg expansion to this compartment and our preliminary observations using peri-ocular or subconjunctiva delivery support such a strategy (CL, VLP, RBL). In total, we posit future studies across multiple types of SOTs may take advantage of Cy based platforms to generate combinatorial strategies for long-term tolerance induction based on PTCy + Treg suppression of inflammation, neovascularization and T cell responses. Addition of compounds to this platform, such as bromodomain inhibitors which diminish inflammatory cytokines and inhibit neovascularization (39, 63) may be particularly worthwhile.

## Data Availability Statement

The original contributions presented in the study are included in the article/Supplementary Material, further inquiries can be directed to the corresponding author/s.

## Ethics Statement

The animal study was reviewed and approved by UM IACUC.

## Author Contributions

CL, DW, SC, and BP designed and performed experiments and wrote the manuscript. YW and HB performed experiments. CB read and edited the manuscript. KK provided support for the research. VP helped design experiments, provided research support, edited, and helped write the manuscript. RL designed experiments, wrote the manuscript, and provided research support. All authors contributed to the article and approved the submitted version.

## Conflict of Interest

RL is a compensated consultant/advisory board member for and equity holder in Heat Biologics. RL and VP received sponsored research funding from Heat Biologics, Inc. The remaining authors declare that the research was conducted in the absence of any commercial or financial relationships that could be construed as a potential conflict of interest.

## References

[1] Luznik L Bolanos-Meade J Zahurak M Chen AR Smith BD Brodsky R. High-dose cyclophosphamide as single-agent, short-course prophylaxis of graft-versus-host disease. Blood. (2010) 115:3224–30. 10.1182/blood-2009-11-251595 20124511 PMC2858487

[2] Luznik L Fuchs EJ. High-dose, post-transplantation cyclophosphamide to promote graft-host tolerance after allogeneic hematopoietic stem cell transplantation. Immunol Res. (2010) 47(1-3):65–77. 10.1007/s12026-009-8139-0 20066512 PMC2892158

[3] Luznik L O'Donnell PV Fuchs EJ. Post-transplantation cyclophosphamide for tolerance induction in HLA-haploidentical bone marrow transplantation. Semin Oncol. (2012) 39:683–93. 10.1053/j.seminoncol.2012.09.005 23206845 PMC3808078

[4] McCurdy SR Kasamon YL Kanakry CG Bolanos-Meade J Tsai HL Showel MM. Comparable composite endpoints after HLA-matched and HLA-haploidentical transplantation with post-transplantation cyclophosphamide. Haematologica. (2017) 102:391–400. 10.3324/haematol.2016.144139 27846611 PMC5286947

[5] Nunes NS Kanakry CG. Mechanisms of graft-versus-host disease prevention by post-transplantation cyclophosphamide: an evolving understanding. Front Immunol. (2019) 10:2668. 10.3389/fimmu.2019.02668 31849930 PMC6895959

[6] Mayumi H Good RA. Long-lasting skin allograft tolerance in adult mice induced across fully allogeneic (multimajor H-2 plus multiminor histocompatibility) antigen barriers by a tolerance-inducing method using cyclophosphamide. J Exp Med. (1989) 169:213–38. 10.1084/jem.169.1.213 2642528 PMC2189174

[7] Berenbaum MC Brown IN. Prolongation of homograft survival in mice with single doses of cyclophosphamide. Nature. (1963) 200:84. 10.1038/200084a0 14074645

[8] Newman DK Isaacs JD Watson PG Meyer PA Hale G Waldmann H. Prevention of immune-mediated corneal graft destruction with the anti-lymphocyte monoclonal antibody, CAMPATH-1H. Eye (Lond). (1995) 9(Pt 5):564–9. 10.1038/eye.1995.140 8543073

[9] Cunnusamy K Paunicka K Reyes N Yang W Chen PW Niederkorn JY. Two different regulatory T cell populations that promote corneal allograft survival. Invest Ophthalmol Vis Sci. (2010) 51:6566–74. 10.1167/iovs.10-6161 20702818 PMC3055769

[10] Leventhal JR Elliott MJ Yolcu ES Bozulic LD Tollerud DJ Mathew JM. Immune reconstitution/immunocompetence in recipients of kidney plus hematopoietic stem/facilitating cell transplants. Transplantation. (2015) 99:288–98. 10.1097/TP.0000000000000605 25594553

[11] Kanakry CG Ganguly S Zahurak M Bolanos-Meade J Thoburn C Perkins B. Aldehyde dehydrogenase expression drives human regulatory T cell resistance to posttransplantation cyclophosphamide. Sci Transl Med. (2013) 5:211ra157. 10.1126/scitranslmed.3006960 24225944 PMC4155575

[12] Ganguly S Ross DB Panoskaltsis-Mortari A Kanakry CG Blazar BR Levy RB. Donor CD4^+^ Foxp3^+^ regulatory T cells are necessary for posttransplantation cyclophosphamide-mediated protection against GVHD in mice. Blood. (2014) 124:2131–41. 10.1182/blood-2013-10-525873 25139358 PMC4186542

[13] Wachsmuth LP Patterson MT Eckhaus MA Venzon DJ Gress RE Kanakry CG. Post-transplantation cyclophosphamide prevents graft-versus-host disease by inducing alloreactive T cell dysfunction and suppression. J Clin Invest. (2019) 129:2357–73. 10.1172/JCI124218 30913039 PMC6546453

[14] Copsel SN Malek TR Levy RB. Medical Treatment Can Unintentionally Alter the Regulatory T-Cell Compartment in Patients with Widespread Pathophysiologic Conditions. Am J Pathol. (2020) 190:2000–12. 10.1016/j.ajpath.2020.07.012 32745461 PMC7527858

[15] Ross D Jones M Komanduri K Levy RB. Antigen and lymphopenia-driven donor T cells are differentially diminished by post-transplantation administration of cyclophosphamide after hematopoietic cell transplantation. Biol Blood Marrow Transplant. (2013) 19:1430–8. 10.1016/j.bbmt.2013.06.019 23819914 PMC5367157

[16] Report of the organ transplant panel. Corneal transplantation. Council on Scientific Affairs. JAMA. (1988) 259:719–22. 10.1001/jama.259.5.719 3275820

[17] Gain P Jullienne R He Z Aldossary M Acquart S Cognasse F. Global Survey of Corneal Transplantation and Eye Banking. JAMA Ophthalmol. (2016) 134:167–73. 10.1001/jamaophthalmol.2015.4776 26633035

[18] Ciba Foundation. Corneal Graft Failure. Amsterdam, NY: Elsevier (1973). viii, 363 p.

[19] Niederkorn JY. Immunology and immunomodulation of corneal transplantation. Int Rev Immunol. (2002) 21(2-3):173–96. 10.1080/08830180212064 12424842

[20] Coster DJ Williams KA. Management of high-risk corneal grafts. Eye (Lond). 2003;17:996–1002. 10.1038/sj.eye.6700634 14631407

[21] Dana MR Streilein JW. Loss and restoration of immune privilege in eyes with corneal neovascularization. Invest Ophthalmol Vis Sci. (1996) 37:2485–94. 8933765

[22] Wolf D Barreras H Bader CS Copsel S Lightbourn CO Pfeiffer BJ. Marked *in vivo* donor regulatory T cell expansion *via* interleukin-2 and TL1A-Ig stimulation ameliorates graft-versus-host disease but preserves graft-versus-leukemia in recipients after hematopoietic stem cell transplantation. Biol Blood Marrow Transplant. (2017) 23:757–66. 10.1016/j.bbmt.2017.02.013 28219835 PMC5625339

[23] Khan SQ Tsai MS Schreiber TH Wolf D Deyev VV Podack ER. Cloning, expression, and functional characterization of TL1A-Ig. J Immunol. (2013) 190:1540–50. 10.4049/jimmunol.1201908 23319737

[24] Chinen T Kannan AK Levine AG Fan X Klein U Zheng Y. An essential role for the IL-2 receptor in Treg cell function. Nat Immunol. (2016) 17:1322–33. 10.1038/ni.3540 27595233 PMC5071159

[25] Cooke KR Kobzik L Martin TR Brewer J Delmonte J Jr Crawford JM. An experimental model of idiopathic pneumonia syndrome after bone marrow transplantation: I. The roles of minor H antigens and endotoxin. Blood. (1996) 88:3230–9. 10.1182/blood.V88.8.3230.bloodjournal8883230 8963063

[26] Tan Y Cruz-Guilloty F Medina-Mendez CA Cutrufello NJ Martinez RE Urbieta M. Immunological disruption of antiangiogenic signals by recruited allospecific T cells leads to corneal allograft rejection. J Immunol. (2012) 188:5962–9. 10.4049/jimmunol.1103216 22593618

[27] Streilein JW McCulley J Niederkorn JY. Heterotopic corneal grafting in mice: a new approach to the study of corneal alloimmunity. Invest Ophthalmol Vis Sci. (1982) 23:489–500. 6749749

[28] Amescua G Collings F Sidani A Bonfield TL Rodriguez JP Galor A. Effect of CXCL-1/KC production in high risk vascularized corneal allografts on T cell recruitment and graft rejection. Transplantation. (2008) 85:615–25. 10.1097/TP.0b013e3181636d9d 18347542

[29] Sano Y Ksander BR Streilein JW. Murine orthotopic corneal transplantation in high-risk eyes. Rejection is dictated primarily by weak rather than strong alloantigens. Invest Ophthalmol Vis Sci. (1997) 38:1130–8. 9152232

[30] Kruisbeek AM Shevach E Thornton AM. Proliferative assays for T cell function. Curr Protoc Immunol. (2004) Chapter 3:Unit 3 12. 10.1002/0471142735 18432927

[31] Wolf D Bader CS Barreras H Copsel S Pfeiffer BJ Lightbourn CO. Superior immune reconstitution using Treg-expanded donor cells versus PTCy treatment in preclinical HSCT models. JCI Insight. (2018) 3:e121717. 10.1172/jci.insight.121717 30333311 PMC6237457

[32] Kanakry CG O'Donnell PV Furlong T de Lima MJ Wei W Medeot M. Multi-institutional study of post-transplantation cyclophosphamide as single-agent graft-versus-host disease prophylaxis after allogeneic bone marrow transplantation using myeloablative busulfan and fludarabine conditioning. J Clin Oncol. (2014) 32:3497–505. 10.1200/JCO.2013.54.0625 25267759 PMC4209101

[33] Kanakry CG Bolanos-Meade J Kasamon YL Zahurak M Durakovic N Furlong T. Low immunosuppressive burden after HLA-matched related or unrelated BMT using posttransplantation cyclophosphamide. Blood. (2017) 129:1389–93. 10.1182/blood-2016-09-737825 28049637 PMC5345732

[34] Boisgerault F Liu Y Anosova N Dana R Benichou G. Differential roles of direct and indirect allorecognition pathways in the rejection of skin and corneal transplants. Transplantation. (2009) 87:16–23. 10.1097/TP.0b013e318191b38b 19136886 PMC2698296

[35] Yamagami S Dana MR Tsuru T. Draining lymph nodes play an essential role in alloimmunity generated in response to high-risk corneal transplantation. Cornea. (2002) 21:405–9. 10.1097/00003226-200205000-00014 11973391

[36] Bachmann B Taylor RS Cursiefen C. Corneal neovascularization as a risk factor for graft failure and rejection after keratoplasty: an evidence-based meta-analysis. Ophthalmology. (2010) 117:1300–5.e7. 10.1016/j.ophtha.2010.01.039 20605214

[37] Inomata T Mashaghi A Di Zazzo A Lee SM Chiang H Dana R. Kinetics of angiogenic responses in corneal transplantation. Cornea. (2017) 36:491–6. 10.1097/ICO.0000000000001127 28060028 PMC5334361

[38] Toomer KH Yuan X Yang J Dee MJ Yu A Malek TR. developmental progression and interrelationship of central and effector regulatory T cell subsets. J Immunol. (2016) 196:3665–76. 10.4049/jimmunol.1500595 27009492 PMC4868642

[39] Copsel SN Lightbourn CO Barreras H Lohse I Wolf D Bader CS. BET bromodomain inhibitors which permit Treg function enable a combinatorial strategy to suppress GVHD in pre-clinical allogeneic HSCT. Front Immunol. (2018) 9:3104. 10.3389/fimmu.2018.03104 30733722 PMC6353853

[40] Inomata T Hua J Di Zazzo A Dana R. Impaired function of peripherally induced regulatory T cells in hosts at high risk of graft rejection. Sci Rep. (2016) 6:39924. 10.1038/srep39924 28008995 PMC5180229

[41] Atif M Conti F Gorochov G Oo YH Miyara M. Regulatory T cells in solid organ transplantation. Clin Transl Immunology. (2020) 9:e01099. 10.1002/cti2.1099 32104579 PMC7036337

[42] Wachsmuth LP Patterson MT Eckhaus MA Venzon DJ Kanakry CG. optimized timing of post-transplantation cyclophosphamide in MHC-haploidentical murine hematopoietic cell transplantation. Biol Blood Marrow Transplant. (2020) 26:230–41. 10.1016/j.bbmt.2019.09.030 31586477 PMC7590501

[43] Jones JW Brody GL Oneal RM Haines RF. Prolongation of skin homografts in rabbits, using cyclophosphamide. J Surg Res. (1963) 3:189–98. 10.1016/S0022-4804(63)80057-8 13964754

[44] Kersey JH Kruger J Song C Kloster B. Prolonged bone marrow and skin allograft survival after pretransplant conditioning with cyclophosphamide and total lymphoid irradiation. Transplantation. (1980) 29:388–91. 10.1097/00007890-198005000-00008 6990564

[45] Cardoso SS Philippens KM von Mayersbach H. The effect of cyclophosphamide upon mitoses in the cornea of rats, a circadian dependent effect. Eur J Cancer. (1978) 14:1037–41. 10.1016/0014-2964(78)90058-0 710475

[46] Cursiefen C Chen L Dana MR Streilein JW. Corneal lymphangiogenesis: evidence, mechanisms, and implications for corneal transplant immunology. Cornea. (2003) 22:273–81. 10.1097/00003226-200304000-00021 12658100

[47] Browder T Butterfield CE Kraling BM Shi B Marshall B O'Reilly MS. Antiangiogenic scheduling of chemotherapy improves efficacy against experimental drug-resistant cancer. Cancer Res. (2000) 60:1878–86. 10766175

[48] Shlomchik WD Couzens MS Tang CB McNiff J Robert ME Liu J. Prevention of graft versus host disease by inactivation of host antigen-presenting cells. Science. (1999) 285:412–5. 10.1126/science.285.5426.412 10411505

[49] Wolf D Schreiber TH Tryphonopoulos P Li S Tzakis AG Ruiz P. Tregs expanded *in vivo* by TNFRSF25 agonists promote cardiac allograft survival. Transplantation. (2012) 94:569–74. 10.1097/TP.0b013e318264d3ef 22902792

[50] Tahvildari M Omoto M Chen Y Emami-Naeini P Inomata T Dohlman TH. *In vivo* expansion of regulatory T cells by low-dose interleukin-2 treatment increases allograft survival in corneal transplantation. Transplantation. (2016) 100:525–32. 10.1097/TP.0000000000001044 26881788 PMC4764457

[51] Shao C Chen Y Nakao T Amouzegar A Yin J Tahvildari M. Local delivery of regulatory T cells promotes corneal allograft survival. Transplantation. (2019) 103:182–90. 10.1097/TP.0000000000002442 30247445 PMC6309927

[52] Lutsiak ME Semnani RT De Pascalis R Kashmiri SV Schlom J Sabzevari H. Inhibition of CD4(+)25 + T regulatory cell function implicated in enhanced immune response by low-dose cyclophosphamide. Blood. (2005) 105:2862–8. 10.1182/blood-2004-06-2410 15591121

[53] Zhao J Cao Y Lei Z Yang Z Zhang B Huang B. Selective depletion of CD4^+^ CD25^+^ Foxp3^+^ regulatory T cells by low-dose cyclophosphamide is explained by reduced intracellular ATP levels. Cancer Res. (2010) 70:4850–8. 10.1158/0008-5472.CAN-10-0283 20501849

[54] Le DT Jaffee EM. Regulatory T-cell modulation using cyclophosphamide in vaccine approaches: a current perspective. Cancer Res. (2012) 72:3439–44. 10.1158/0008-5472.CAN-11-3912 22761338 PMC3399042

[55] Winzler C Fantinato M Giordan M Calore E Basso G Messina C. CD4(+) T regulatory cells are more resistant to DNA damage compared to CD4(+) T effector cells as revealed by flow cytometric analysis. Cytometry A. (2011) 79:903–11. 10.1002/cyto.a.21132 22015731

[56] Bayer AL Jones M Chirinos J de Armas L Schreiber TH Malek TR. Host CD4^+^ CD25^+^ T cells can expand and comprise a major component of the Treg compartment after experimental HCT. Blood. (2009) 113:733–43. 10.1182/blood-2008-08-173179 18832651 PMC2628379

[57] Bayer AL Chirinos J Cabello C Yang J Matsutani T Malek TR. Expansion of a restricted residual host Treg-cell repertoire is dependent on IL-2 following experimental autologous hematopoietic stem transplantation. Eur J Immunol. (2011) 41:3467–78. 10.1002/eji.201141611 21928285 PMC3516388

[58] Fang L Adkins B Deyev V Podack ER. Essential role of TNF receptor superfamily 25 (TNFRSF25) in the development of allergic lung inflammation. J Exp Med. (2008) 205:1037–48. 10.1084/jem.20072528 18411341 PMC2373837

[59] Nishikii H Kim BS Yokoyama Y Chen Y Baker J Pierini A. DR3 signaling modulates the function of Foxp3^+^ regulatory T cells and the severity of acute graft-versus-host disease. Blood. (2016) 128:2846–58. 10.1182/blood-2016-06-723783 27760760 PMC5159706

[60] Ito S Bollard CM Carlsten M Melenhorst JJ Biancotto A Wang E. Ultra-low dose interleukin-2 promotes immune-modulating function of regulatory T cells and natural killer cells in healthy volunteers. Mol Ther. (2014) 22:1388–95. 10.1038/mt.2014.50 24686272 PMC4089007

[61] Hirakawa M Matos TR Liu H Koreth J Kim HT Paul NE. Low-dose IL-2 selectively activates subsets of CD4(+) Tregs and NK cells. JCI Insight. (2016) 1:e89278. 10.1172/jci.insight.89278 27812545 PMC5085610

[62] Yu A Snowhite I Vendrame F Rosenzwajg M Klatzmann D Pugliese A. Selective IL-2 responsiveness of regulatory T cells through multiple intrinsic mechanisms supports the use of low-dose IL-2 therapy in type 1 diabetes. Diabetes. (2015) 64:2172–83. 10.2337/db14-1322 25576057

[63] Huang M Qiu Q Xiao Y Zeng S Zhan M Shi M. BET bromodomain suppression inhibits VEGF-induced angiogenesis and vascular permeability by blocking VEGFR2-mediated activation of PAK1 and eNOS. Sci Rep. (2016) 6:23770. 10.1038/srep23770 27044328 PMC4820704

