# Supplementary figures and images for: Use of Post-transplant Cyclophosphamide Treatment to Build a Tolerance Platform to Prevent Liquid and Solid Organ Allograft Rejection
Source: Front Immunol. 2021 Mar 2;12:636789. doi: 10.3389/fimmu.2021.636789 (PMC7962410; doi:10.3389/fimmu.2021.636789)

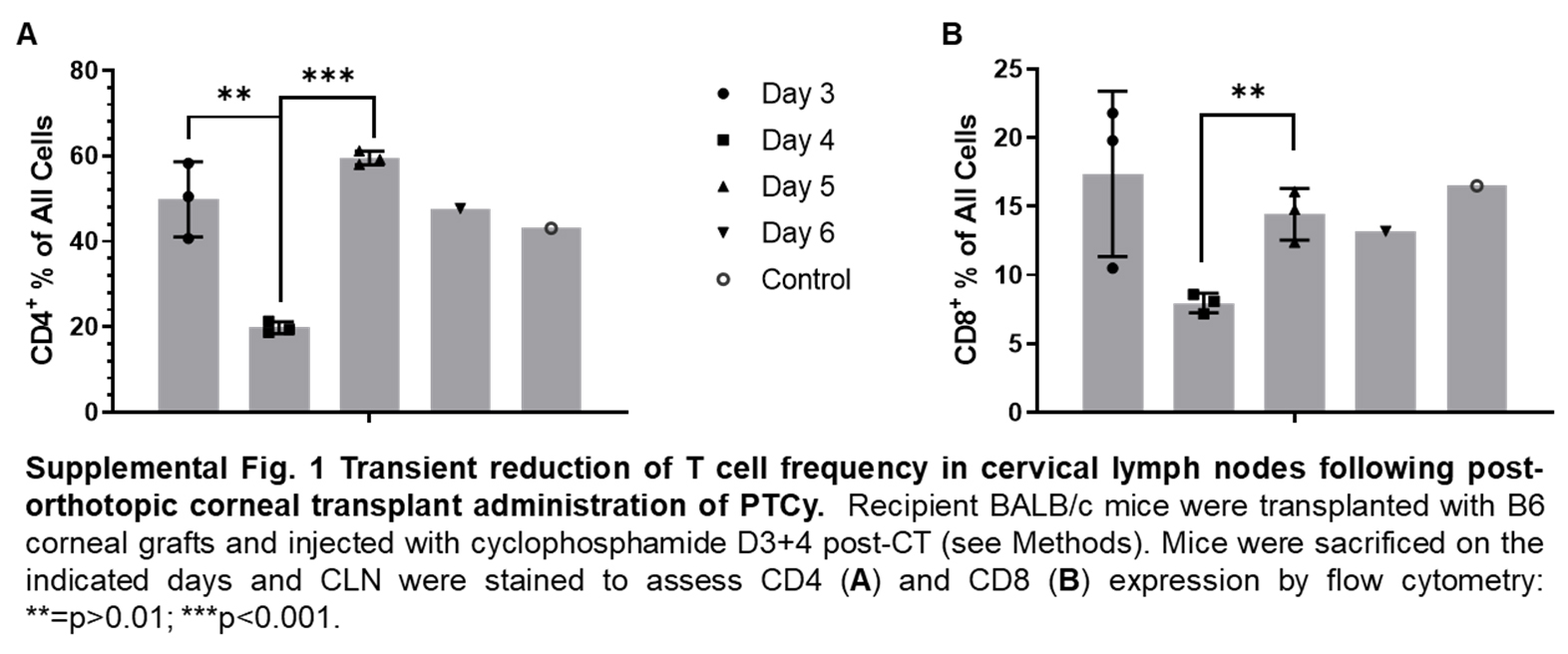

Supplement: Supplementary file 1 [file Image_1.JPEG]

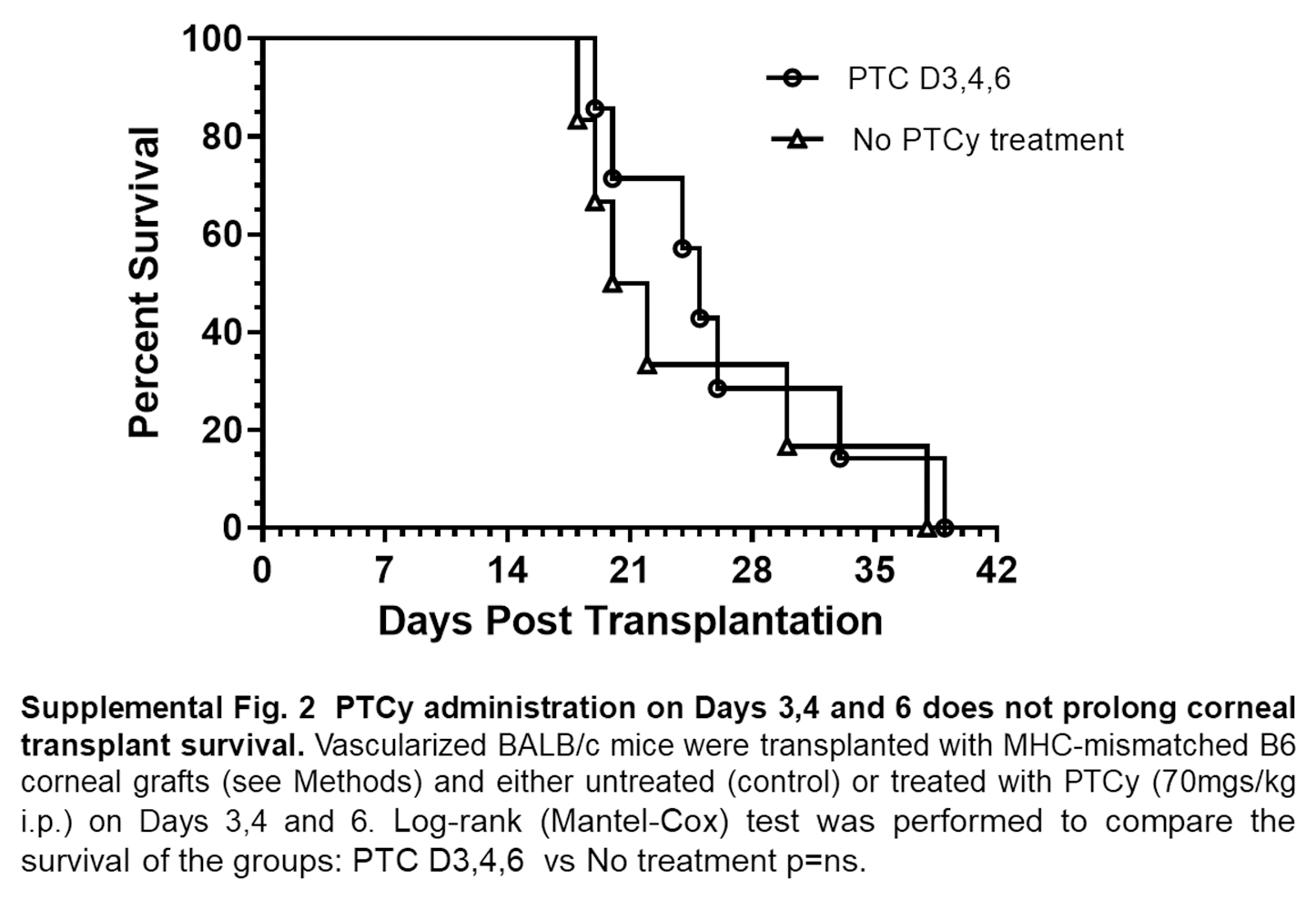

Supplement: Supplementary file 2 [file Image_2.JPEG]

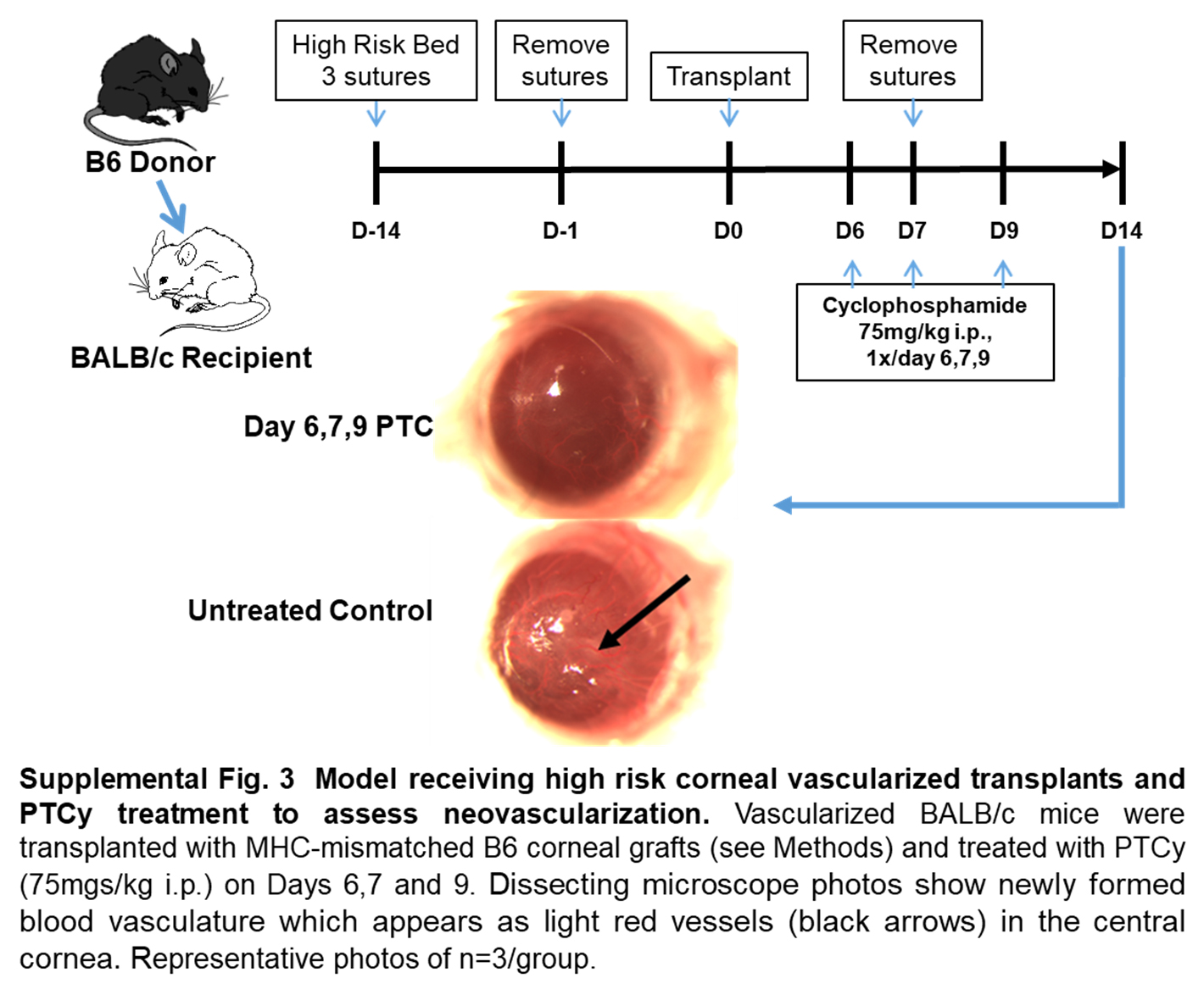

Supplement: Supplementary file 3 [file Image_3.JPEG]

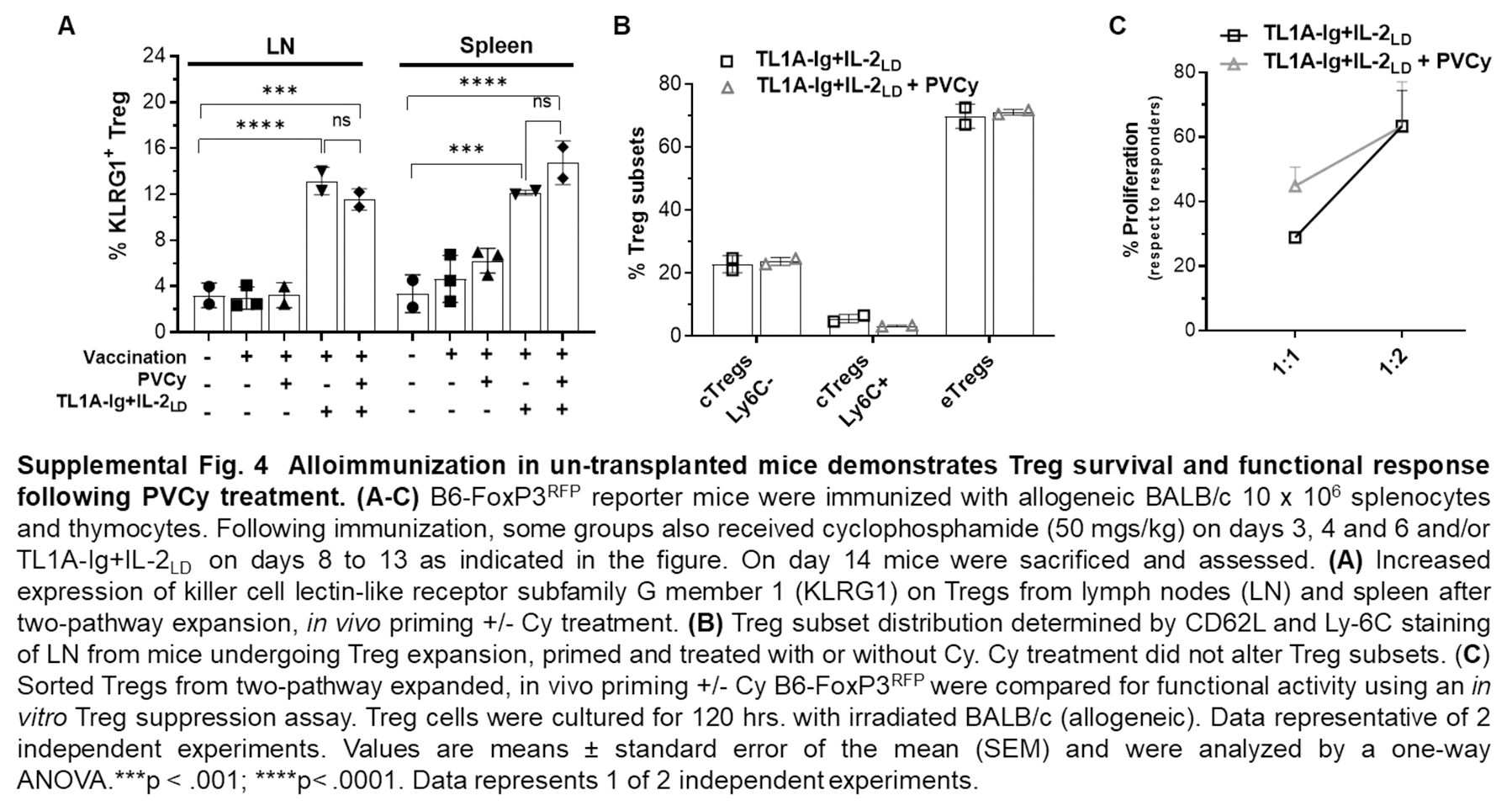

Supplement: Supplementary file 4 [file Image_4.JPEG]
